# An Integrated Optofluidic Platform Enabling Total Phosphorus On-Chip Digestion and Online Real-Time Detection

**DOI:** 10.3390/mi11010059

**Published:** 2020-01-01

**Authors:** Chang Li, Bingbing Wang, Hao Wan, Rongxiang He, Qi Li, Siyuan Yang, Wencan Dai, Ning Wang

**Affiliations:** 1 National Engineering Laboratory for Fiber Optic Sensing Technology, Wuhan University of Technology, Wuhan 430070, China; lichang@whut.edu.cn (C.L.); wanhao@whut.edu.cn (H.W.); yangsiyuan@whut.edu.cn (S.Y.); dwc0102@whut.edu.cn (W.D.); 2 Wuhan Safety & Environmental Protection Research Institute, Sinosteel Group Co., Ltd, Wuhan 430081, China; 602797303@163.com; 3 Institute for Interdisciplinary Research & Key Laboratory of Optoelectronic Chemical Materials and Devices of Ministry of Education, Jianghan University, Wuhan 430056, China; herx@jhun.edu.cn; 4 Wuhan Space Sanjiang LITRI Co., Ltd, Wuhan 430075, China; whutyj@163.com

**Keywords:** optofluidic device, total phosphorus, on-chip digestion, real-time detection, hollow optical fiber

## Abstract

This paper presents a total phosphorus online real-time monitoring system integrated with on-chip digestion based on the merits of optofluidic technology. The integrated optofluidic device contains a hollow optical fiber employed for pretreatment and digestion of phosphorus solution samples, a polydimethylsiloxane (PDMS)-based micromixer with convergent–divergent walls designed to enable sufficient mixing and chromogenic reaction, and a couple of optical fiber collimators attached with a Z-shaped flow cell for optical detection. Details of system design and fabrication are introduced in this paper. In the experiment, on-chip digestion of four typical phosphates in aqueous solution including organophosphorus and inorganic phosphorus is investigated under different reaction conditions, such as digestion temperature, concentration of oxidant and pH value, and the optimal reaction parameters are explored under different conditions. Meanwhile, we demonstrate the online real-time monitoring function of the optofluidic device, and the digestion mechanisms of four different phosphates are analyzed and discussed. Compared with the national standard method, we find that the measurement accuracy and sensitivity are acceptable when the concentration of total phosphorus is between 0.005–0.9 mg/L (by weight of P) in aqueous solution, which covers the range defined in the national standard. The traditional digestion time of several hours is greatly reduced to less than 10 s, and the content of total phosphorus can be obtained in a few minutes. The integrated optofluidic device can significantly shorten the test time and reduce the sample amount, and also provides a versatile platform for the real-time detection and analysis of many biochemical samples.

## 1. Introduction

Phosphorus is one of the indispensable nutrients for all kinds of natural water. However, excessive phosphorus in water will cause eutrophication and seriously deteriorate the ecological environment [1,2,3]. Therefore, monitoring of total phosphate in surface water, sewage and industrial wastewater has become a focus all over the world. Various methods have been tried in recent years, such as electrochemical methods, chromatography, mass spectrometry, optical detection methods and so on [4,5]. Here, as one of the most popular optical methods, UV-visible (UV-Vis) spectrophotometry has been employed for the Chinese national standard method (Water-Quality-Determination of total phosphorus-ammonium molybdate spectrophotometric method (GB 11893-89)) because of its simple detection mechanism [6]. This method usually accompanies with complex processes involving digestion, chromogenic reaction, absorption spectrum detection and harsh pretreatment such as processing in high-pressure sterilizer with high temperature. Moreover, it is usually time-consuming, and the equipment employed is bulky with complex operation, high energy consumption and low efficiency.

Recently, due to its versatility, optofluidic technology has been involved in many fields [7,8]. Among them, its application to water quality monitoring and detection has attracted extensive attention, including detection of heavy metal ions [9], microorganisms [10], inorganic salts [11] and so on. In recent years, online detection of phosphorus has attracted attention, and researchers have tried to make a breakthrough using optofluidic technology. For example, Tong et al. designed a photocatalytic microreactor for phosphorus digestion in 2015 [12]. The TiO_2_ film was immobilized in a microreactor irradiated by UV light for photocatalytic digestion of the phosphorus samples. However, for the non-flowing system, it required extraction of the digested phosphorus samples first from the chip and then measurement of them with a UV-Visible spectrometer, in which the phosphorus samples were easily influenced by the environment and the complex operation, resulting in many measurement errors. What is more, the photocatalytic microreactor presented low digestion efficiency. After that, an optofluidic system integrated with an optical fiber Fabry–Pérot resonator was set up for phosphate detection by Zhu et al. in 2017 [5], which could quickly perform the chromogenic reaction of orthophosphate and reduce the detection time to less than 10 s and the design of the microcavity could improve detection limitation and accuracy. Nevertheless, except for orthophosphate, most phosphorus in nature cannot be induced into a chromogenic reaction directly, and usually accompanying pretreatment is needed. Further, it is difficult to implement the alignment of optical fibers in the microchannel. Based on the above understanding, the greatest challenge for the design of an integrated optofluidic system for monitoring phosphorus is building on-chip high-temperature and high-pressure surroundings with oxidant, integration of various function and alignment of optical components.

In this work, an integrated optofluidic platform enabling on-chip pretreatment of phosphorus samples and online real-time monitoring their concentrations is designed and set up, including a digestion cell, chromogenic reaction cell and Z-shaped flow cell with optical fiber collimators (OFC) for absorption spectrum testing. Because gas bubbles generate easily in the microchannels for most microchips based on polydimethylsiloxane (PDMS) and poly(methylmethacrylate) (PMMA), especially under high temperature conditions, this affects the subsequent chromogenic reaction and optical absorption measurements. A hollow optical fiber with a core diameter of 100 µm is employed herein to build a high-temperature and high-pressure environment that prevents gas bubble issues. In order to integrate it on the microchip and enlarge its utilization efficiency, the hollow optical fiber is coiled to coin-sized spirals for the phosphorus digestion reaction. Then, a micromixer with convergent–divergent walls are designed for efficient mixing and chromogenic reaction [13,14]. For the part of optical absorption measurement, a Z-shaped flow cell allocated with a couple of optical fiber collimators is fabricated, in which the length of the flow cell is approximately 1 cm to be the same as the light path, abiding by the Beer–Lambert law. Here, the optical fiber collimators ensure an easier alignment and operation. In the experiment, we explore the optimal digestion conditions including the temperature, pH and concentration of the oxidant for four typical phosphorous salts in surface water and analyze their digesting mechanism. In addition, we simulate the fluid mixing processes of samples in the microchannels and verify the integrated optofluidic device’s functions and performance of the on-chip digestion and online real-time monitoring of total phosphorus. This detection system is suitable for on-chip pretreatment and online monitoring of most of the biochemical samples and the successful demonstration will showcase the versatility of the integrated optofluidic platform.

## 2. Design and Principle

### 2.1. Device Design

**Figure 1:**
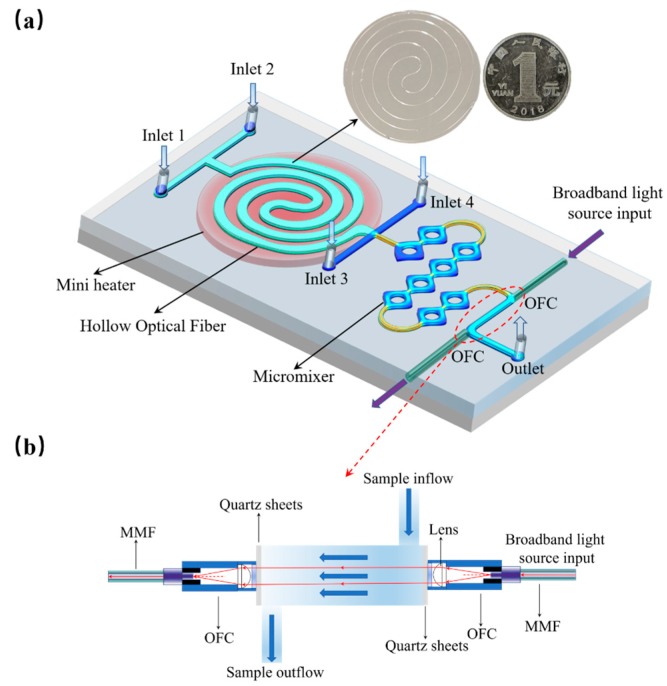
Design of the integrated optofluidic device: (**a**) 3D schematic of the device. The inner diameter of the hollow optical fiber for digestion is 100 µm and the height of mixer is 150 µm; (**b**) the design and cross section of the part for optical detection. The lens of the optical fiber collimator has a focal length of 10 mm, and a wavelength range of 650–1050 nm. The spot size of the spot of the output light is about 0.6 mm, and its numerical aperture (NA) of the multimode optical fiber (MMF) is 0.22 (chrome-plated brass fiber with core diameter of 200 µm).

The design and 3D diagram of the integrated optofluidic device are shown in Figure 1a. It mainly consists of three functional parts: The first one is a spiral hollow optical fiber (Figure 1a inset) with the total length of 27 cm and inner diameter of 100 µm as a microchannel for the phosphorus digestion reaction; the second part is a PDMS-based micromixer for the chromogenic reaction; finally, the optical detection part is composed by a Z-shaped flow cell and a couple of optical fiber collimators. All the above functional parts are fixed on a glass slide (7.5 cm × 3 cm). A miniature heater (3 cm × 3 cm) is placed under the spiral hollow optical fiber to construct a high temperature and high pressure surrounding for phosphorus digestion reaction. When the aqueous phosphorus samples and oxidant are simultaneously injected into the hollow optical fiber in proportion by a syringe pump, the heater supplies enough heat quantity and pressure for the digestion reaction while avoiding gas bubble generation. For the part of chromogenic reaction, chromogenic agent A (ascorbic acid solution) and chromogenic agent B (ammonium molybdate solution, concentrated sulfuric acid and antimony potassium tartrate solution) are injected into the micromixer and reacts with the digested samples. Here, the micromixer is designed with typical convergent–divergent walls with the microchannel height of 150 µm [14]. Finally, the chromogenic samples are transferred to the Z-shaped flow cell for optical absorption detection.

The details and cross section of the Z-shaped flow cell are shown in Figure 1b, which is mainly constructed by PDMS-based microchannel with the dimension of 10 mm (L) × 1.5 mm (W) × 1.5 mm (H), and a couple of optical fiber collimators output with a multimode optical fiber (MMF) pigtail embedded on both sides of the flow cell [15]. The fabrication details are similar to our previous work [6]. Here, the length of flow cell and light path is designed as 1 cm by the Beer-Lambert law, and the outer diameter of the OFC is 1 mm for its assembly and easily alignment. Between the solution samples and the OFC, there were two quartz sheets (5 mm × 5 mm × 100 µm) inserted to avoid the influence on the optical absorption test.

### 2.2. Materials and Instruments

Potassium persulfate (K_2_SO_4_), sodium hydroxide (NaOH), sodium glycerophosphate (C_3_H_7_Na_2_O_6_P), sodium tripolyphosphate (Na_5_P_3_O_10_), tetrasodium pyrophosphate (Na_4_P_2_O_7_) and disodium guanosine 5′-monophosphate (C_10_H_12_N_5_Na_2_O_8_P) were purchased from Sinopharm Chemical Reagent Co. (Shanghai, China); the details of the 4 phosphates are shown in Table 1 Polydimethylsiloxane (PDMS, DC184) was purchased from Dow Corning Co. (Midland, MI, USA). Ascorbic acid solution, ammonium molybdate, concentrated sulfuric acid and antimony potassium tartrate were purchased from Sinopharm Chemical Reagent Co. (Shanghai, China), and used to prepare the chromogenic agent A and B. The syringe pump was purchased from Longer Precision Pump Co. (LSP02-2B, Baoding, China). The UV-Vis spectrometer and the halogen lamp is purchased from Avantes technology Co. (AvaSpec-ULS2048L, Apeldoorn, The Netherlands). The fiber collimators are customized from YiHao communication technology co., Ltd (Wuhan, China).

### 2.3. Fabrication of the Optofluidic Device

Firstly, the hollow optical fiber for phosphorus digestion reaction was spirally coiled fixed on a glass side by UV-curable adhesive on a glass slide as shown in Figure 1a inset. Then, the microchannels for the chromogenic reaction and the Z-shaped flow cell were fabricated by the standard photolithography [6]. Before molding the PDMS-based microchannel, the sites for fixing the OFC had also been designed and reserved in advance. On both sides of the flow cell along the direction of light path, there were two small holes with larger size than the OFCs for fixing them. When performing the alignment, both of a couple of OFCs were nipped on 5D optical stages, and one was connected with the light source and the other one is jointed with an optical power meter. Once the alignment was conducted to obtain the maximum optical power displayed on power meter, the holes were sealed by UV- adhesive and PDMS, and the OFCs were fixed and released.

### 2.4. Calibration of the Optofluidic Device

**Figure 2:**
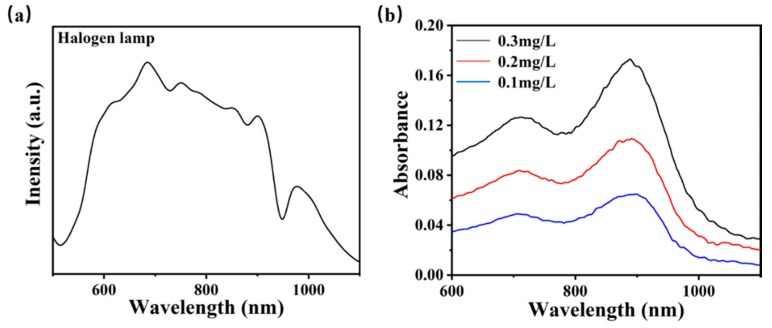
Characterization of light source and pre-validation of chromogenic reaction; (**a**) spectrum of the halogen lamp as the broadband light source; (**b**) the absorbance of the pre-validation solution converted by orthophosphate samples with different concentrations.

When measuring the phosphate in water, the phosphate is usually converted into PMo heteropoly blue by the chromogenic reaction, which has a strong characteristic absorption peak at about 890 nm [5]. First, the light source used for the detection is calibrated and measured by spectrometer. Its spectrum is shown in Figure 2a with a broadband wavelength range of 600–900 nm and spanned the characteristic absorption peak of PMo heteropoly blue [16]. The power of the equilibrium halogen lamp is 10 W and the output optical power of pigtail is about 17 µW and the variation of light intensity was measured to less than 2% in 1 h.

Before the experiment, we conducted the pre-experiment. Three potassium phosphate monobasic (KH_2_PO_4_) solution samples with different concentrations were directly pumped in the device and converted to PMo heteropoly blue, and then the optical transmission was measured and transferred to the absorbance plotted in Figure 2b. There are two absorption peaks in the spectrum at 882 nm and 700 nm, and the absorption at 882 nm were chosen as the characteristic absorption peaks related to the concentration changes of PMo heteropoly blue solutions.

### 2.5. Online Monitoring of Phosphate by the Optofluidic Device

In the experiment of phosphate online digestion and online real-time monitoring, sodium glycerophosphate (C_3_H_7_Na_2_O_6_P), sodium tripolyphosphate (Na_5_P_3_O_10_), tetrasodium pyrophosphate (Na_4_P_2_O_7_) and disodium guanosine 5′-monophosphate (C_10_H_12_N_5_Na_2_O_8_P) were employed as the typical phosphate solution samples, and their initial concentration was set as 0.3 mg/L (by weight of P). Ascorbic acid (C_6_H_8_O_6_) was used to prepare the chromogenic agent A. Meanwhile, ammonium molybdate [(NH_4_)_6_Mo_7_O_24_·4H_2_O)], potassium antimony tartrate (KSbC_4_H_4_O_7_·1/2H_2_O) and H_2_SO_4_ were used to prepare the chromogenic agent B. Potassium persulfate (K_2_S_2_O_8_) was utilized as digesting oxidant [12,17,18]. Various parameters affecting the performance of the optofluidic platform were investigated, such as digestion temperature (controlled by the heater), pH values (adjusted by adding H_2_SO_4_/NaOH solutions) and the initial concentration of the K_2_S_2_O_8_ solution.

## 3. Experimental Results

### 3.1. Simulation of the Flowing in the Micromixer

**Figure 3:**
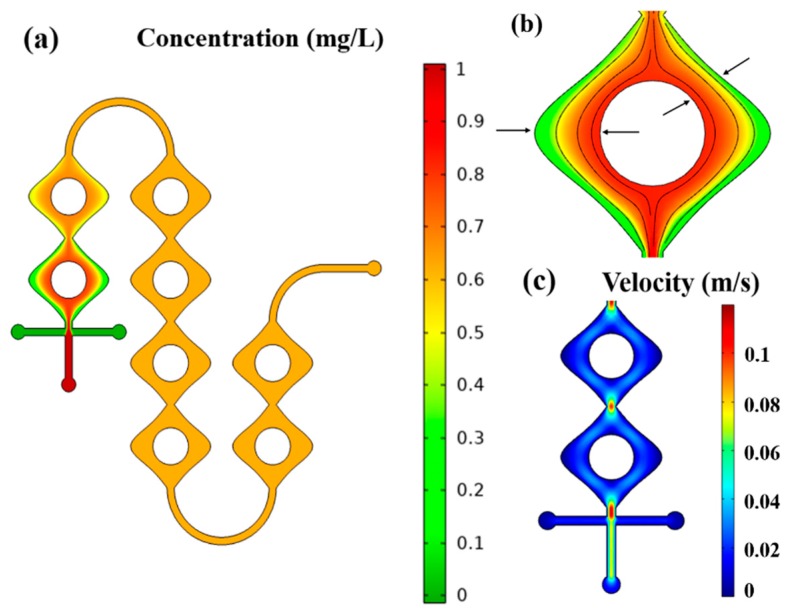
Simulation of flow for mixing and chromogenic reaction; (**a**) concentration distribution in the mixer; (**b**) distortion of concentration lamina; (**c**) flow rate distribution in the mixer.

In order to demonstrate the effective mixing of fluids in the microreactor, COMSOL Multiphysics^®^ (5.4, COMSOL, Inc., Stockholm, Sweden) was employed to simulate and obtain the flow in the micromixer. During the mixing process, we set the initial velocity at the inlet as 0.083 m/s (50 µL/min) and 0.025 m/s (15 µL/min). As shown in Figure 3a, the main microchannel was first divided into two sub-channels and then reconfigured at periodic intervals along the length of the microchannel. The sub-channels were provided with sinusoidally varying convergent–divergent walls. The outer wall and the center circle exert centripetal and centrifugal forces on the liquid as it flows through it, which caused more distortion of concentration lamina and faster mixing at various cross-sectional planes of the microchannel, as shown in Figure 3b [13,14]. The microflow was also accelerated at the throat of each converging–diverging cell to further improve the performance of the micromixer as in Figure 3c. Moreover, it was found that the mixing was nearly completed when flowing into the second cell. Therefore, the design of convergent–divergent wall could ensure the thoroughly mix and chromogenic reaction before the optical detection.

### 3.2. Calibration of Total Phosphorus

To calibrate the performance of the optofluidic device for online real-time detecting the total phosphorus, the Beer-Lambert law [19,20] is utilized to obtain the absorbance and concentration changes of PMo heteropoly blue, which could be expressed as follows:

(1) $${A=\log}_{10}\;(I_{0}/I)=kcl$$

where *A* is absorbance, *I* and *I*_0_ are the light intensity of incident light and transmission light, respectively, *k* is the molar absorptivity with units of L/(mol·cm), *c* is the concentration of the compound in solution, expressed in mol/L, *l* is the path length of the sample in the flow cells.

**Figure 4:**
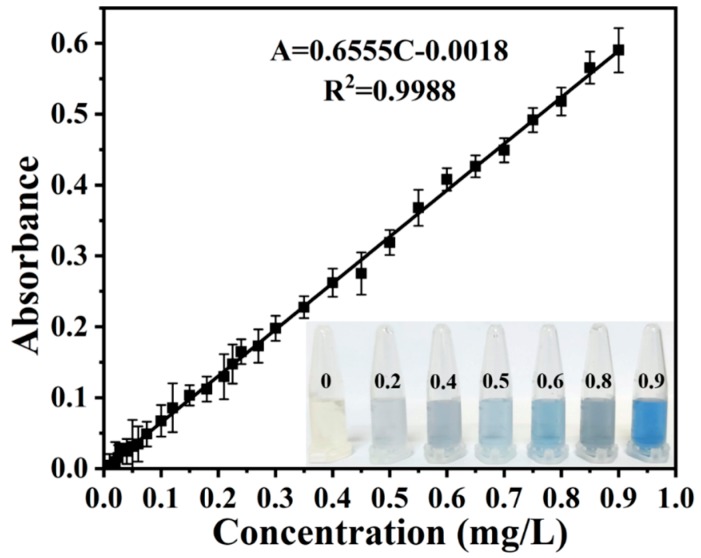
Calibration and standard curve of concentration of total phosphorus as function of absorbance; inset shows the color of the pre-validation solution samples converted with orthophosphate with different concentrations by the chromogenic reaction.

In the experiment, 32 standard phosphate solution samples with different concentrations (potassium dihydrogen phosphate) were firstly prepared ranging from 0.005 to 0.9 mg/L, which covered the range of the national standard. Before the chromogenic reaction, both the chromogenic agent A and chromogenic agent B were injected at the flow rate of 15 µL/min to carry out absorbance baseline to avoid the experimental errors. After that, the different standard phosphate solution samples were pumped in at the flow rate of 50 µL/min for the chromogenic reaction and were converted to PMo heteropoly blue for optical absorption measurement. The absorbance as the function of the concentrations of standard phosphate solution samples are plotted in Figure 4 and the inset shows the different PMo heteropoly blue samples converted by typical standard phosphate solutions with relative concentrations. Each data point was repeated for three times and the error bar represents the standard deviation of the mean. The standard curve presents a good linearity and could match best with the following equation:

(2) A=0.6555c−0.0018

where *A* is the absorbance of the blue complex formed by the chromogenic reaction after deducting the background, and *c* is the concentration of total phosphorus solution samples. When measuring the amount of phosphates in aqueous samples, we can obtain the real digestion rate through the standard curve and the absorbance. Results suggest that the designed optofluidic device can be used for measuring the concentration of total phosphorus in aqueous samples.

### 3.3. The Overall Performance of Optofluidic Device

#### 3.3.1. Effect of Temperature

**Figure 5:**
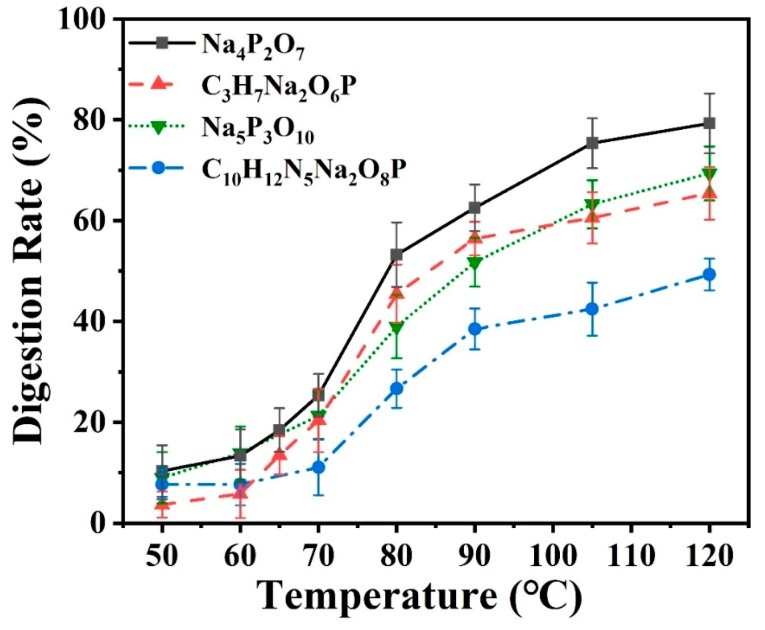
Online measurement of the digestion efficiency of four kinds of phosphate solution samples at different temperatures; the concentration of K_2_SO_4_ was prepared as 50 g/L and the pH value was 11.4.

**Figure 6:**
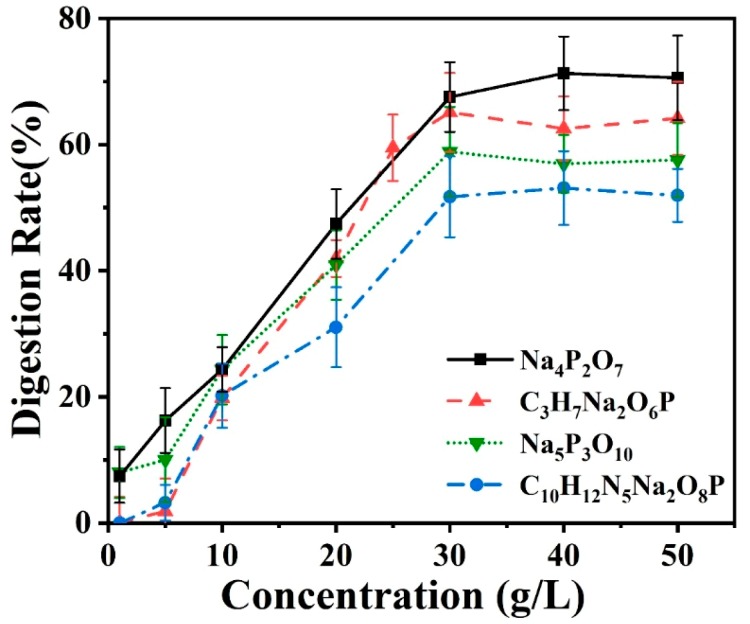
Online measurement of the digestion efficiency of four kinds of phosphate solution samples under different concentrations of the oxidant at the digestion temperature of 90 °C and pH value of 11.4.

**Figure 7:**
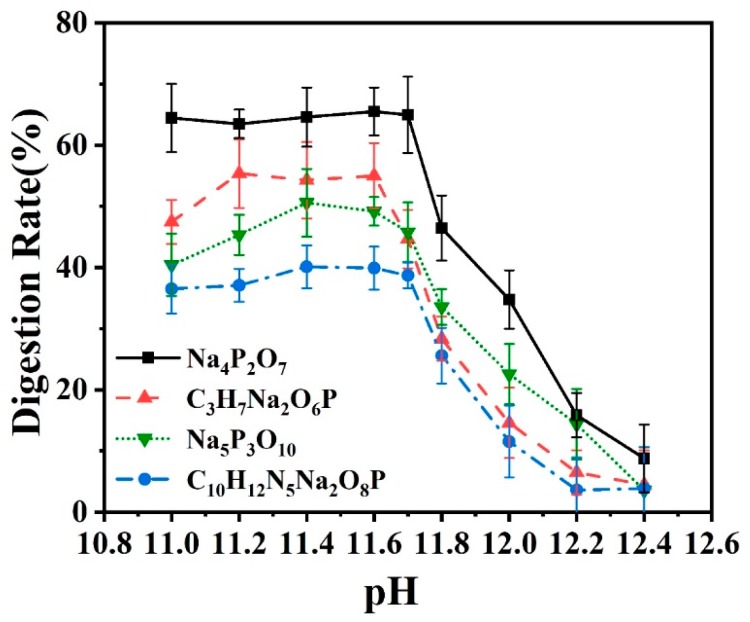
Online measurement of the digestion efficiency of four kinds of phosphate solution samples under different pH values at the digestion temperature of 90 °C and the concentration of K_2_SO_4_ was prepared as 50 g/L.

Temperature was an important factor affecting the phosphate digestion processes. It affected the activity of oxidant and caused gas bubbles generated easily in microchannels especially at a higher temperature which not only influenced the flow and reaction, but also the following on-chip optical detection. If the bubbles enter the Z-shaped flow cell, it will cause huge error for optical detection. Thus, the effect of temperature was investigated in the experiment. A miniheater was placed under spiral microchannel of the digestion to adjust the reaction temperature, and the different typical phosphates solution samples including inorganic phosphate and organic phosphate with the same concentration of 0.3 mg/L (by weight of P) were prepared and pumped into the optofluidic device with the flow rate of 50 µL/min [5]. Meanwhile, the concentration of K_2_SO_4_ was prepared as 50 g/L and the flow rate of K_2_SO_4_ and chromogenic agent A and B was set as 15 µL/min. Before recording the spectrum, we usually waited for about 10 min and used an infrared thermometer to calibrate the temperature of the hollow optical fiber for ensuring the stable reaction and detection. Each data point was repeated for three times, the error bar represents the standard deviation of the mean of the three data points. We calculated the concentration of orthophosphates in Figure 5, Figure 6 and Figure 7 according to the standard curve (Figure 4) obtained in Section 3.2. The experimental results were plotted in Figure 5. The digestion temperature was adjusted and controlled between 50 °C and 120 °C. It was found that K_2_SO_4_ lost its oxidizing activity and all phosphate nearly remained undigested when the temperature was set below 60 °C. With the increase of temperature, the digestion rate of the four kinds of phosphate rises sharply and reaches the highest rate at 120 °C, which indicated that the effect of temperature on the digestion was very large. The digestion rate of tetrasodium pyrophosphate was the highest, followed by sodium glycerophosphate and sodium tripolyphosphate, and that of disodium guanosine 5′-monophosphate was the lowest. The details for the digestion mechanism will be given later. However, when the temperature exceeded 100 °C, gas bubbles were occasionally generated in the microchannel [21]. Therefore, for this kind of optofluidic device, it is better to keep the reaction temperature at 90 °C.

#### 3.3.2. Effect of Concentration of the Oxidant

The initial concentration of the oxidant could affect the digestion of phosphate. In the microreactor, the amount of oxidant was a trace amount, and the digestion time was relatively short, and incomplete digestion reaction might exist in the device. Therefore, it is essential to explore the best concentration of oxidant. Based on the optimal reaction temperature obtained in the last section, the digestion concentration of the oxidant was investigated by using different concentrations of K_2_SO_4_ between 0–50 g/L while controlling the reaction temperature at 90 °C. In this experiment, all the involved reagents were pumped into device at the same flow rate mentioned in the last section. The experimental results are shown in Figure 6. Each data point was repeated for three times, and the error bar represented the standard deviation of the mean. For all the phosphates employed, the digestion rate increased with the increase of the initial concentration of K_2_SO_4_. The digestion rate of tetrasodium pyrophosphate was still the highest, followed by sodium glycerophosphate and sodium tripolyphosphate, and disodium guanosine 5′-monophosphate was still relatively difficult to digest. When the concentration of oxidant was adjusted larger than 30 g/L, the digestion rate reached the maximum and tended towards saturation. This was because the concentration of potassium persulfate was sufficient for the digestion reaction in the optofluidic device, however, in this event, the digestion was still an incomplete digestion which might be limited by the reaction temperature and reaction time. However, compared with the traditional digestion reactors, the reaction time has been immensely shortened to less than 10 s.

#### 3.3.3. Effect of pH Value

The pH value was also a key parameter to influence the digestion reaction, and the oxidant in particular presented higher oxidation activity under alkaline conditions. In the experiment, H_2_SO_4_ and NaOH were employed to adjust the pH value of the initial phosphate solution, while the temperature was set at 90 °C and the initial oxidant concentration was prepared as 50 g/L. All reagents involved were set as the same flow rates referred in Section 3.3.1. When the pH value of the reaction system was adjusted between 9–11, phosphorus existed mostly in the form of HPO_4_^2−^, which easily produced more H_3_PO_4_. However, when the pH value was adjusted to larger than 11, the content of PO_4_^3−^ would increase in the reaction system, which was more conducive to the chromogenic reaction. Therefore, in this experiment, we adjusted the pH value of the reaction system to between 11–12.4. Each data point was repeated for three times, and the error bar represented the standard deviation of the mean. As seen from the results plotted in Figure 7, when pH values were adjusted between 11–11.7, the digestion for all four kinds of phosphates presented a constant reaction rate. However, when pH value was larger than 11.7, all the digestion rates dropped sharply, becoming lower than 10% at pH = 12.4. Tetrasodium pyrophosphate still showed the largest digestion rate in the exploration of pH value effects.

#### 3.3.4. Discussion for the Digestion Reaction

According to the above experimental studies of phosphate digestion under different conditions, the tetrasodium pyrophosphate always presented the highest digestion rate among the four typical phosphates, followed by sodium glycerophosphate and sodium tripolyphosphate, and the disodium guanosine 5′-monophosphate seemed to be the most difficult one to digest. That result might be related to their molecular structures and the breaking of covalent bonds, which were shown in Table 1 by red dash lines.

The digestion of phosphate was essentially an oxidation reaction. Under alkaline conditions, the oxidant K_2_SO_4_ solution could generate hydroxyl radicals when heated to a specific temperature, which would oxidize and convert the four typical phosphates to the orthophosphates for the further chromogenic reaction. It can be seen from the experimental results that the digestion of inorganic phosphorus was easier to achieve than that of organic phosphorus. For the inorganic polyphosphate, tetrasodium pyrophosphate and sodium tripolyphosphate have similar molecular structures, but the former has lower polymerization degree and was easy to hydrolyze to orthophosphate under heating conditions. Therefore, heating an oxidant can simultaneously promote its digestion, resulting in the best digestion efficiency.

However, as for the two organic phosphates, the digestion rate of sodium glycerophosphate was higher than that of disodium guanosine 5′-monophosphate. This was because the steric hindrance effect of sodium glycerophosphate was lower, and the two hydroxyl hydrogen bonds in its structure made the O–C bond more easily disconnected and promoted the oxidation of phosphate ions. Disodium guanosine 5′-monophosphate had complex molecular structure, a high steric hindrance effect and more difficult oxidation reaction, and the amino and purine ring oxidation sites in its structure would also consume the oxidants in the reagents. Therefore, the digestion rate of disodium guanosine 5′-monophosphate would be comparatively lower when adding the same amount of oxidants.

## 4. Discussion

The optofluidic device integrates a high-temperature and high-pressure reaction chamber for pretreatment, a micromixer for chromogenic reaction and a flow cell for optical absorption, which can be used for rapid on-chip pretreatment, and detection of various biochemical samples and reactions to quantify their efficiency and optimize the operating conditions. In this kind of optofluidic detection system, comprehensive data on biochemical samples can be obtained less than 10 s, which is much faster than other bulky reactors which often take several hours or more time by traditional methods. It also avoids the complex pretreatment, sampling, cleaning and measurement, as the fluid sample can self-refresh the flow cell by itself efficiently. Therefore, this kind of optofluidic device is especially suitable for the rapid analysis and characterization of biochemical reactions that need rapid pretreatment of samples of trace amount, rather than other on-site sampling, laboratory pretreatment and measurement technologies.

It is worth mentioning that there may be a small amount of molecular adsorption and microbubble adsorption on the inner wall of the flow cell, especially under the high temperature and high pressure environment, which may affect the reaction and optical path test, and bring the measurement errors. Therefore, it is necessary to control the temperature and normalize the cleaning processes, so as to improve the repeatability and accuracy of the measurement. Beyond that, we used one-factor-at-a-time (OFAT) optimization testing of the reaction conditions (digestion temperature, concentration of oxidant, pH) in this experimental exploration, and thus our testing ran the risk of only finding a local optimum. OFAT experiments cannot tease out interactions between the variables, as a factorial approach might uncover.

In addition, the optical detection part utilizes a couple of commercial optical fiber collimators, which can easily be adjusted and installed. Although its precision and sensitivity may not be comparable with that of gas chromatography (detection limit: 10^−5^ mg/L), it does omit many complex and expensive optical elements. In fact, the measurement range and sensitivity can meet the needs of most detection by optical absorption measurements.

## 5. Conclusions

In conclusion, an integrated optofluidic platform enabling the total phosphorus on-chip digestion and online real-time monitoring was designed and fabricated, which was constructed with a digestion functional cell, chromogenic reaction cell and Z-shaped flow cell combined with optical fiber collimators for optical absorption measurement. When exploring the different factors affecting on the digestion of total phosphorus in the optofluidic platform, the digestion temperature, initial concentration of the oxidant and pH value were investigated. When the reaction temperature was fixed at 90 °C, the concentration of oxidant prepared to be 30 g/L and the pH value adjusted to be between 11–11.7, the maximum digestion rate of tetrasodium pyrophosphate could reach up to about 75%. The different digestion mechanism of four different phosphates were also analyzed and discussed. The integrated optofluidic device can conduct a rapid pretreatment and detection of total phosphorus in 10–20 s and obtain the analysis result in a few minutes. Meanwhile, the detection ranges between 0.005–0.9 mg/L and the detection accuracy in this work is acceptable and covers the Chinese national standard. Combined with the advantages of short-time pretreatment and trace amount of samples, this kind of integrated optofluidic system could provide a versatile platform for the study of on-chip pre-reaction and online real-time analysis of various biochemical samples.

## Figures and Tables

**Table 1:** Details of the four typical phosphates.

Molecular Name	Molecular Formula	Molecular Weight	Molecular Structure
Sodium Glycerophosphate	C_3_H_7_Na_2_O_6_P	216.04	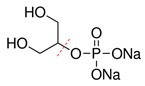
Disodium Guanosine 5′-Monophosphate	C_10_H_12_N_5_Na_2_O_8_P	407.18	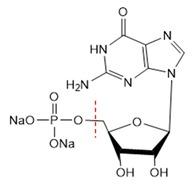
Tetrasodium Pyrophosphate	Na_4_P_2_O_7_	265.9	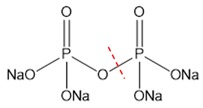
Sodium Tripolyphosphate	Na_5_P_3_O_10_	367.86	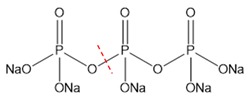

## References

[1] Doney S.C. (2010). The growing human footprint on coastal and open-ocean biogeochemistry. Science.

[2] Wang N., Tan F., Tsoi C.C., Zhang X. (2017). Photoelectrocatalytic microreactor for seawater decontamination with negligible chlorine generation. Microsyst. Technol..

[3] Khan F.A., Ansari A.A. (2005). Eutrophication: An ecological vision. Bot. Rev..

[4] Huang H., Zhang P., Zhang Z., Liu J., Xiao J., Gao F. (2016). Simultaneous removal of ammonia nitrogen and recovery of phosphate from swine wastewater by struvite electrochemical precipitation and recycling technology. J. Clean. Prod..

[5] Zhu J.M., Shi Y., Zhu X.Q., Yang Y., Jiang F.H., Sun C.J., Zhao W.H., Han X.T. (2017). Optofluidic marine phosphate detection with enhanced absorption using a Fabry-Pérot resonator. Lab Chip.

[6] Wang N., Tan F., Zhao Y., Tsoi C.C., Fan X., Yu W., Zhang X. (2016). Optofluidic UV-Vis spectrophotometer for online monitoring of photocatalytic reactions. Sci. Rep..

[7] Psaltis D., Quake S.R., Yang C. (2006). Developing optofluidic technology through the fusion of microfluidics and optics. Nature.

[8] Kar A.K. Photofluidics—A new platform for biophotonics. Proceedings of the 2012 International Conference on Fiber Optics and Photonics.

[9] Cook A.M., Daughton C.G., Alexander M. (1978). Determination of phosphorus-containing compounds by spectrophotometry. Anal. Chem..

[10] Chinnasamy S., Bhatnagar A., Hunt R.W., Das K.C. (2010). Microalgae cultivation in a wastewater dominated by carpet mill effluents for biofuel applications. Bioresour. Technol..

[11] Villagrán C., Deetlefs M., Pitner W.R., Hardacre C. (2004). Quantification of halide in ionic liquids using ion chromatography. Anal. Chem..

[12] Tong J., Dong T., Bian C., Wang M., Wang F., Bai Y., Xia S. (2015). An integrated photocatalytic microfluidic platform enabling total phosphorus digestion. J. Micromech. Microeng..

[13] Lee C.Y., Wang W.T., Liu C.C., Fu L.M. (2016). Passive mixers in microfluidic systems: A review. Chem. Eng. J..

[14] Afzal A., Kim K.Y. (2012). Passive split and recombination micromixer with convergent-divergent walls. Chem. Eng. J..

[15] Wang N., Dai T., Lei L. (2018). Optofluidic technology for water quality monitoring. Micromachines.

[16] Yan Y., Yang Q., Wang J., Jin H., Wang J., Yang H., Zhou Z., Tian Q., Yang S. (2017). Heteropoly blue doped polymer nanoparticles: An efficient theranostic agent for targeted photoacoustic imaging and near-infrared photothermal therapy in vivo. J. Mater. Chem. B.

[17] Hosomi M., Sudo R. (1986). Simultaneous determination of total nitrogen and total phosphorus in freshwater samples using persulfate digestion. Int. J. Environ. Stud..

[18] Maher W., Krikowa F., Wruck D., Louie H., Nguyen T., Huang W.Y. (2002). Determination of total phosphorus and nitrogen in turbid waters by oxidation with alkaline potassium peroxodisulfate and low pressure microwave digestion, autoclave heating or the use of closed vessels in a hot water bath: Comparison with Kjeldahl digestion. Anal. Chim. Acta.

[19] Swinehart D. (1962). The beer-lambert law. J. Chem. Educ..

[20] Parnis J.M., Oldham K.B. (2013). Beyond the beer-lambert law: The dependence of absorbance on time in photochemistry. J. Photochem. Photobiol. A Chem..

[21] Zheng W., Wang Z., Zhang W., Jiang X. (2010). A simple PDMS-based microfluidic channel design that removes bubbles for long-term on-chip culture of mammalian cells. Lab Chip.

